# Aroma Compound Changes in the *Jiangxiangxing* Baijiu Solid-State Distillation Process: Description, Kinetic Characters and Cut Point Selection

**DOI:** 10.3390/foods13020232

**Published:** 2024-01-11

**Authors:** Yuchen Gao, Qiang Yang, Guangyuan Jin, Shengzhi Yang, Ruiyang Qin, Linjie Lyu, Xianze Yao, Rongzhen Zhang, Shuang Chen, Yan Xu

**Affiliations:** 1 Lab of Brewing Microbiology and Applied Enzymology, Key Laboratory of Industrial Biotechnology of Ministry of Education, State Key Laboratory of Food Science & Technology, School of Biotechnology, Jiangnan University, Wuxi 214122, China; 13546456265@163.com (Y.G.); g.jin@jiangnan.edu.cn (G.J.); 6220201092@stu.jiangnan.edu.cn (R.Q.); rzzhang@jiangnan.edu.cn (R.Z.); shuangchen@jiangnan.edu.cn (S.C.); 2 Jing Brand Co., Ltd., Huangshi 435100, China; 00504@jingpai.com (Q.Y.); 01097@jingpai.com (S.Y.); 05167@jingpai.com (L.L.); 16432@jingpai.com (X.Y.)

**Keywords:** baijiu, solid-state distillation, aroma compounds, kinetic character, cut points

## Abstract

Solid-state distillation is a distinctive process for extracting the baijiu aroma compounds that determine the flavor character of baijiu. In this study, the changes in various chemical properties of the aroma compounds in three classical *Jiangxiangxing* baijiu fermented grain distillation processes were analyzed. The changes in the aroma compounds in the instantaneous distillates were quantified and correlation analyzes were conducted. The results showed that the effect of the aroma compounds was greater than the differences between the fermented grains. Eleven representative aroma compounds were selected to develop the kinetic models describing two opposing changes. For the regulation of the *Jiangxiangxing* baijiu aroma compounds, their recovery rates were calculated using a kinetic model. A comprehensive comparison of the recovery rates of the characteristic aroma and other aroma compounds at different cut-off values revealed that the optimum recovery rate of the characteristic aroma of *Jiangxiangxing* baijiu 2,3,5,6-tetramethylpyrazine was 14.53% at cut-off values of 3.9 and 19.8 min. In this study, representative changes in the aroma compounds and the selection of cut-off values to regulate the baijiu distillation aroma were proposed.

## 1. Introduction

Hundreds of trace aroma compounds commonly define the flavor character of spirits. The types of aroma of different spirits have distinctive flavor characters. Spirits’ flavor characters form in the distillation process; the distillation process extracts these aroma compounds from fermented grains or fruits [1,2]. During distillation, the aroma compound concentration changes with the distillation time [3]. The selection of distillation time cut points can regulate the spirit aroma compounds’ recovery rate at different concentrations and proportions [4]. Distillers define the distillation time cut points to select the aroma compounds and obtain different aroma properties in spirits [5]. Different criteria are used to define the cut points during distillation, such as ethanol recovery, alcoholic strength or the temperature of the vapor before it enters the condenser [6]. However, regulating spirits’ aroma compounds in distillation mainly depends on the distiller experience and online tasting and smelling techniques [7]. Therefore, analysis of the change in distillates’ aroma compounds can provide the basis for achieving aroma regulation during distillation.

During distillation, three sections are chosen sequentially, head, heart and tail, using two cut point selection according to the distillation time [8]. The cut point selection in distillation depends on knowing the aroma compound change character and change prediction [9]. In liquid-state distillation systems, the aroma compound change has been investigated for predicting and regulating spirit products such as brandy [10,11], whisky [12], Brazilian sugarcane spirits [13] and rum [14]. These studies summarized the aroma compound change characters in liquid-state distillation. Based on the aroma compound change character, some prediction models [15,16] and cut point selection optimal models [7,17] were developed. But for baijiu solid-state distillation, its mass transfer process is different from liquid-state distillation [18]. Baijiu is distilled using solid-state fermented grains: sorghum, rice, wheat, corn and others (mixtures and other starchy plants such as yam are also used) [19]. Liquid-state distillation models cannot be used to predict and regulate baijiu aroma compounds. In the above studies, different aroma markers were analyzed for each distillation cut [9,20]. Several of these aroma compounds, such as ethyl acetate and β-damascenone for *Qingxiangxing* baijiu or 2,3,5,6-tetramethylpyrazine for *Jiangxiangxing* baijiu, play an important role in the formation of the distinctive aroma of the heart [21,22,23]. These markers can improve our understanding of the change in the aroma compounds depending on the distillation time. But our understanding of the characters of these changes is lacking. Therefore, it is necessary to describe the changing characters of aroma compounds and discuss their relationships in order to apply the selection of cut-off points.

Analysis of the aroma compound change in baijiu during distillation can indicate the change character and support a quantization foundation for regulating the aroma compounds in baijiu solid-state distillation. As the typical aroma type for baijiu, *Jiangxiangxing* (JXX) baijiu has great economic value and international influence due to its unique aroma, taste experience and comforting effect post-drinking [24]. In 2022, JXX baijiu saw an annual production of 70 million hectoliters, realized a sales revenue of 29.573 billion dollars and obtained a total profit of 12.252 billion dollars [25]. Its key aroma compound 2,3,5,6-tetramethylpyrazine has significant functional activity [26]. Although it is the typical aroma type for baijiu, there are few studies on the changes in the aroma compounds of *Jiangxiangxing* (JXX) baijiu during distillation. Therefore, the kinetic modeling approach was applied in this paper to investigate the change in the aroma compounds during the solid-state distillation of JXX baijiu. The multi-objective optimization (MOO) model was used to select the optimal cut points. The objectives of this study were (1) to clarify the changes in the aroma compounds with the distillation time of JXX baijiu, (2) to evaluate the differences in the changes in aroma compounds with different properties, (3) to quantify the changes in the aroma compounds using the kinetic model, (4) to select the optimal cut points for regulating the aroma compound recovery proportions using MOO. By clarifying the character of the change in the aroma compounds, the mechanism of the mass transfer of aroma compounds in the solid-state distillation of baijiu can be further discussed. Further, studying the selection of cut points in baijiu solid-state distillation supports further methods to regulate the aroma compounds in baijiu production.

## 2. Materials and Methods

### 2.1. Chemicals

Analytical-grade sodium chloride was purchased from Sinopharm Chemical Reagent Co., Ltd. (Shanghai, China). Ethanol (GC-grade, ≥99% purity) was purchased from J&K Scientific Co., Ltd. (Beijing, China). The Headspace Solid-Phase Microextraction–Gas Chromatography–Mass Spectrometry (HS-SPME–GC–MS) internal standards (ISs) 2,2-dimethyl-propanoic acid (IS1), 2-octanol (IS2), 2-phenylethyl acetate-D_3_ (IS3), n-hexyl-D_13_ alcohol (IS4) and 2-methoxyphenol-D_3_ (IS5) and the Gas Chromatography with Flame Ionization Detection (GC-FID) internal standards (ISs) 2-methylbutan-2-ol (IS1), pentyl acetate (IS2) and 2-ethylbutanoic acid (IS3) were supplied by Sigma-Aldrich Chemicals Co. (Shanghai, China), at a high-purity grade (GC-grade, ≥97% purity). The other chemical standards, including 1,1-diethoxyethane, 1-butanol, 1-hexanol, 1-octanol, 1-pentanol, 1-propanol, 2,3,5,6-tetramethylpyrazine, 2,3,5-trimethylpyrazine, 2-methoxy-4-methylphenol, 2-methylpropanal, 2-methylpropanoic acid, 2-methylpropanol, 2-methylpropyl acetate, 2-phenylethanol, 2-phenylethyl acetate, 3-hydroxybutan-2-one, 3-methylbutanal, 3-methylbutanol, 3-methylbutyl acetate, 4-ethyl-2-methoxyphenol, 4-methylphenol, acetaldehyde, acetic acid, benzaldehyde, butanoic acid, butyl hexanoate, diethyl butanedioate, dimethyl trisulfide, ethyl 3-methylbutanoate, ethyl 3-phenylpropanoate, ethyl acetate, ethyl butanoate, ethyl decanoate, ethyl dodecanoate, ethyl heptanoate, ethyl hexadecanoate, ethyl hexanoate, ethyl lactate, ethyl nonanoate, ethyl octanoate, ethyl oleate, ethyl pentanoate, ethyl phenylacetate, ethyl tetradecanoate, furfural, heptanoic acid, hexanoic acid, hexyl hexanoate, isoamyl butyrate, octan-3-ol, octanoic acid, pentan-2-ol, pentanoic acid, phenol, phenylacetaldehyde, propanoic acid and propyl hexanoate, were purchased from Sigma-Aldrich Chemicals Co. (Shanghai, China). Pure water was obtained using a Milli-Q purification system (Millipore, Bedford, MA, USA).

### 2.2. Sampling

**Figure 1:**
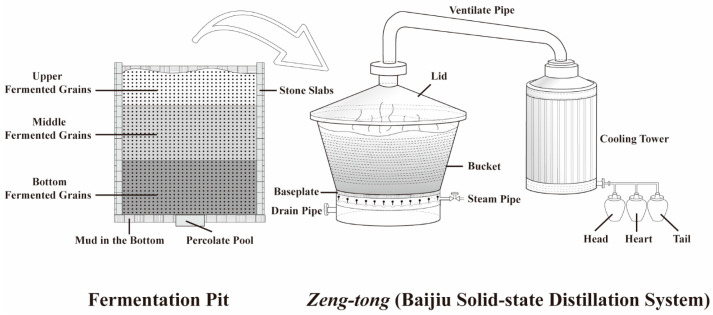
The solid-state distillation process steps and system of fermented grains of baijiu.

The instantaneous distillates of the JXX baijiu distillation had to be examined in order to discuss the change in aroma compounds. The distillation experiment was conducted at Jing Brand Maotai Town Liquor Co., Ltd. (Zunyi, China). Three different fermentation-pit-layer fermented grains (upper fermented grains (called *Mian zao*, MZ), middle fermented grains (called *Zhong zao*, ZZ) and bottom fermented grains (called *Di zao*, DZ), respectively, as shown in Figure 1, were used for distillation. The total fermented grain filling weight was 700–800 kg, while the intake steam pressure during the distillation process was maintained at 1.2–1.5 MPa. The instant distillates were collected every six minutes until the end of the distillation process using a 250 mL glass bottle. For each distillation batch, distillation was carried out for 54 min, and 10 distillate samples were obtained. A total of 60 samples were collected from six batches (MZ, ZZ and DZ were distilled in two batches each) of the JXX baijiu solid-state distillation process. The samples were stored at room temperature (25 °C).

### 2.3. Quantification of the Aroma Compounds

Since there are many types of aroma compounds with different properties and a wide range of concentrations in the distillation samples, two quantification methods (GC-FID and HS-SPME–GC–MS) were used in this study to obtain more accurate quantitative results. The compounds with a higher concentration (more than 100 mg/L) were quantified using the GC-FID technique. Other compounds with a lower concentration were quantified using the GC–MS technique. A total of 57 common baijiu flavoring compounds were quantified in this study [27].

***Quantification using GC-FID techniques.*** GC-FID was employed for the quantitation of certain aroma compounds which had a high concentration. This was carried out using an Agilent 6890 GC equipped with a FID, modified from the method reported by Gao [23]. All analyses were conducted in triplicate. Each sample was adjusted to a volume of 1 mL with an ethanol content of 50% (ABV) using pure water. A total of 1 mL of the saturated sample was put into a 2 mL injection vial and spiked with 50 μL of an internal standard mixed with IS1, 2-methylbutan-2-ol, 111,040 μg/L; IS2, pentyl acetate, 125,500 μg/L and IS3, 2-ethylbutanoic acid, 102,570 μg/L. The column carrier gas was nitrogen at a constant flow rate of 1 mL/min. The separations were performed using a DB-Wax column (30 m length, 0.25 mm i.d., 0.25 μm film thickness; J&W Scientific, Folson, CA, USA) with an oven temperature program of 60 °C (3 min), ramped at 5 °C/min to 150 °C (15 min) and then ramped at 10 °C/min to 230 °C (5 min). One microliter of the diluted liquor sample (50% ethanol by volume) was injected into the GC. The split ratio was 37:1. The injector and detector temperatures were 250 °C. Identification of the compounds and the correction factors calculated in 50% ethanol by volume was performed.

***Quantification using HS-SPME–GC–MS techniques.*** Other aroma compounds were quantitated using HS-SPME-GC-MS as described previously [28]. Each sample was adjusted to a volume of 5 mL with an ethanol content of 10% (ABV) using pure water. A total of 5 mL of the sample, which was saturated with 1.5 g of NaCl, was put into a 20 mL sampler vial and spiked with 40 μL of an internal standard mixed with IS1, 2,2-dimethyl-propanoic acid, 1197 μg/L; IS2, 2-octanol, 294 μg/L; IS3, 2-phenylethyl acetate-D_3_, 146 μg/L; IS4, n-hexyl-D_13_ alcohol, 322 μg/L; IS5, 2-methoxyphenol-D_3_, 307 μg/L. The vial was then sealed with a PTFE/silicone septum and a screw top. All analyses were conducted in triplicate.

An SPME automatic headspace sampling system (CTC Analytics AG, Zwingen, Switzerland) with a 120 μm divinylbenzene/carbon wide range/polydimethylsiloxane (DVB/CAR WR/PDMS) fiber (CTC Analytics AG, Switzerland) was used for extraction and injection. The sample was equilibrated at 45 °C for 5 min and extracted for 45 min at the same temperature under stirring at a rotation speed of 250 rpm. After extraction, the fiber was automatically inserted into the GC injection port (at 250 °C) for 5 min to desorb the analytes. The samples were separated on a DB-FFAP column (60 m × 0.25 mm i.d.; 0.25 µm film thickness; Agilent Technologies, Inc.) in spitless mode. The oven temperature was held at 50 °C for 2 min, raised to 230 °C at a rate of 6 °C/min and held at 230 °C for 15 min. The mass spectrometer was operated in electron ionization mode at 70 eV. The temperature of the ion source was 230 °C. The mass range was set from 35 to 350 amu in full scan mode.

The aroma peaks were identified according to comparison with the mass spectral database of the National Institute of Standards and Technology (NIST) and the retention index (RI, determined using n-alkanes C7–C40) [29]. In addition, each standard stock solution was prepared by dissolving a standard compound in the model solution and then diluting it to 10 different concentrations. The same amount of internal standard as in the samples was added to the standard solutions, and then the working standard solutions were analyzed under the same conditions as the samples. Calibration curves were generated by plotting the ratio of the peak area of the reference compounds to the corresponding internal standard against the concentration ratio. As described previously [30], quantification with internal standards and calibration curves was performed in this study. In addition, these quantification data for all distillation samples were used for further data mining.

### 2.4. Statistical Analysis

Several statistical analyses were used to describe yjr distillation aroma compounds change differences and characters. The heatmap analysis and hierarchical cluster analysis (HCA) were conducted by Yihanbo Biological Information. Principal components analysis (PCA) was carried out using SIMCA version 14.0 (Umetrics, Umeå, Sweden). SPSS (version 19.0, IBM, Inc., Armonk, NY, USA) was used for the Pearson correlation analysis. The figures were created using Origin (version 9.0).

### 2.5. Kinetic Change Rate and Recovery Rate

For calculating the ethanol and aroma compound recovery rate and kinetic character at different distillation cut points, their cumulative amount with distillation time could be calculated using the development of kinetic models. The model assumed the distillates’ flows were maintained consistently across the distillation time.

A pseudo-first-order equation was used to develop the ethanol and aroma compound kinetic models. It was expressed as Equation (1) [31]:

(1) $$C_{t}=C_{e}{(1-e}^{-Kt})$$

where $C_{t}$ was the aroma compounds’ cumulative amount at a certain distillation time (μg/L), $C_{e}$ was the aroma compounds’ cumulative amount at equilibrium (μg/L), K was the distillation constant (1/min) and t was a certain distillation time.

As calculated by Equation (1), the aroma compounds’ cumulative amount at a certain time could be obtained. Therefore, the change rate at a certain moment could be calculated using Equation (1):

(2) $$v=C_{e}Ke^{-Kt}$$

where v is the change rate (μg/(L/min)).

According to the aroma compounds’ kinetic parameters, their recovery rates during distillation could be obtained:

(3) $$r=\frac{C_{t2}-C_{t1}}{C_{tmax}}$$

where r was the recovery rates (%), $t_{1}$ and $t_{2}$ represented two distillation cut points and $t_{max}$ was the maximum distillation time (the maximum distillation time was 54 min in this experiment).

### 2.6. Multi-Objective Optimization (MOO)

The multi-objective cost function considered the JXX baijiu’s aroma characters. Quality was associated with the objective aroma compound composition in the heart cut, i.e., low concentrations of the head and tail, as well as high objective aroma compound concentrations of the heart. The process productivity was defined by the ethanol recovery in the heart cut.

The multi-objective optimization problem can be formulated as follows [7]:

(4) $$Min\;F\left(x_{i}\right)=\left[{\;f}_{1}\left(x_{i}\right),\;f_{2}\left(x_{i}\right),\;\ldots \;,\;f_{n}\left(x_{i}\right)\right]$$

subject to

$$\frac{{dy}_{n}\;}{dt}=z\left(y_{n},\;x_{i},\;t\right),\;n=1,\;2,\ldots ,\;N\;g_{j}\left(x_{i}\right)\geq \;0,\;j=1,\;2,\ldots \;,\;J\;h_{k}\left(x_{i}\right)=0,k=1,2,\ldots ,K\;x_{i}^{L}\;\leq \;x_{i}\;\leq \;x_{i}^{U}\;,\;i=1,\;2,\;\ldots ,\;I$$

where $f\;(x_{i})$ represents the objectives that must be minimized simultaneously, $z(y_{n},\;x_{i},\;t)$ represents the differential equations of the model, $g(x_{i})$ represents the inequality constraints, $h(x_{i})$ represents the equality constraints and $x_{i}$ the decision variables.

## 3. Results and Discussion

### 3.1. Aroma Compound Change in JXX Baijiu Solid-State Distillation

**Figure 2:**
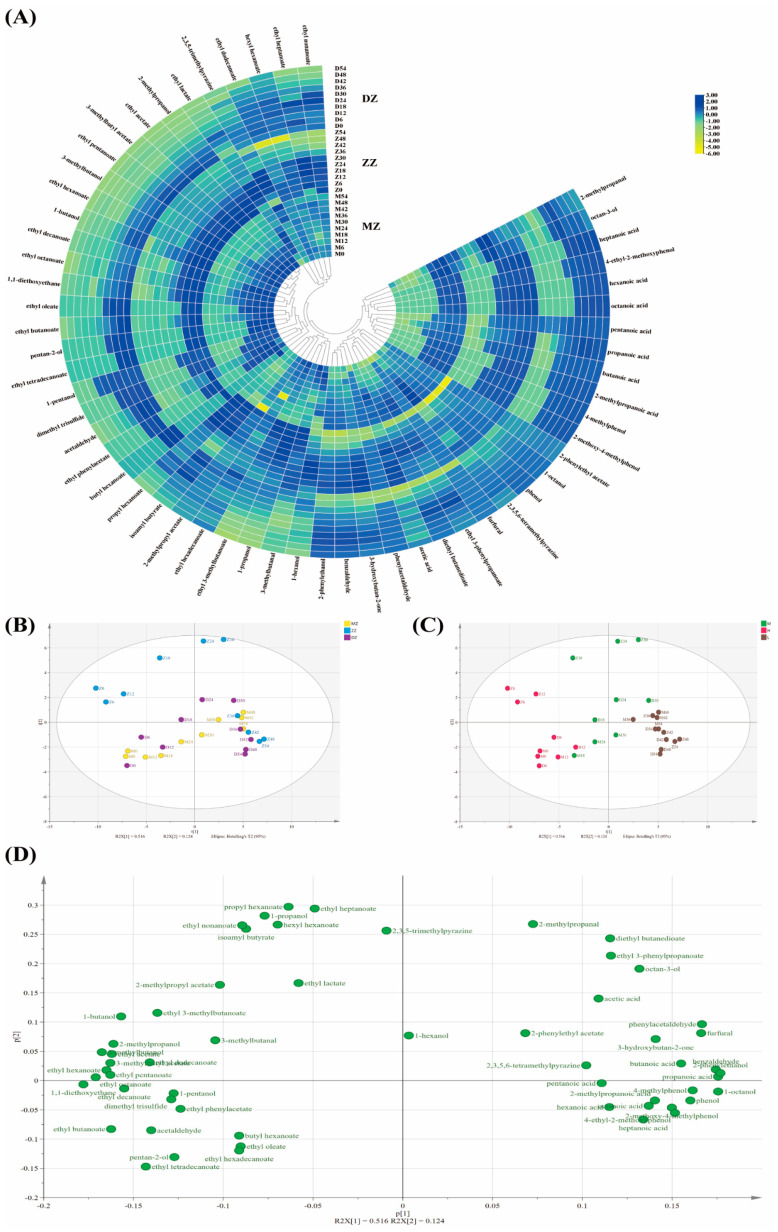
(**A**) Heatmaps of aroma compound changes in distillation with different fermentation-pit-layer fermented grains (MZ, ZZ and DZ). (**B**) PCA score scatter plots which were classed by fermented conditions. (**C**) PCA score scatter plots which were classed by alcohol content (H (ABV > 50%), M (50% > ABV > 10%), L (ABV < 10%)). (**D**) PCA loading plot which described aroma compound distribution.

**Analysis of aroma compound distribution in JXX baijiu solid-state distillation.** To illustrate the changes in the MZ, ZZ and DZ distillation due to the quantitative aroma compounds, the concentrations of the quantitative compounds in the 10 distillates were used to create a heatmap. The color (from yellow to blue) indicates the relative concentration, which changes from low to high. First, a Z-score normalization transformation was performed for all aroma compounds before the heatmap was created. The differences between the aroma compounds at different distillation times are shown in Figure 2A. The HCA clearly assigned the quantitative markers in all three different fermentation layers of the fermented grain distillation to two clusters. Cluster I showed a significantly lower mean concentration in the early distillates and showed an increasing trend with extended distillation time. Cluster II showed a decreasing trend with extended distillation time. The circular heatmap and the HCA analysis therefore contradicted the changes between the individual aroma compounds, as these compounds were more soluble in water and had a higher boiling point. In the early stage of distillation, a large amount of ethanol was evaporated, which was not conducive to the distillation of these water-soluble compounds. In the later stage, a large amount of water-soluble and high-boiling-point compounds were distilled and accumulated in the tail as the ethanol concentration decreased [32]. This phenomenon led to difficulties in determining the cutting times to balance the aroma compounds and obtain baijiu with different aroma characteristics [18].

Based on the aroma compound data, PCA was performed to determine the distribution of the samples; the scatter plots of the PCA points are shown in Figure 2B,C. The samples were classified according to the conditions of the fermentation pit layers (MZ, ZZ and DZ) and the alcohol content (H (ABV > 50%), M (50% > ABV > 10%) and L (ABV < 10%), respectively. A trend of intergroup separation was shown on the PCA plot for the data in both the positive and negative groups. The baijiu samples clustered into three distinct groups based on the t1 × t2 score. The results of the calculation were a R^2^X value of 79.3% and a prediction ability parameter (Q^2^) of 61.2%. The PCA1 was 80.7% and the PCA2 was 17.33%. The fermented condition groups did not cluster well (Figure 2B). Therefore, differences in the fermented conditions had little effects on the changes in the aroma compounds. The aroma compound concentration change tendencies were not affected by the fermentation conditions. The changes in the aroma compounds could be divided by the alcohol content (Figure 2C). These results showed that significant differences were present between the JXX baijiu samples at different distillation times, although significant differences were not present between the distillates of different groups obtained under different fermented conditions. Therefore, in same solid-state distillation systems, the distillation changes in the aroma compounds were only affected by the mass transfer principle of the distillation system. The different pit layer fermentation conditions only affected the aroma compound concentration. The aroma compound concentration change tendencies were not affected by the fermentation conditions.

Further information can be taken from the loading plot (Figure 2D). Here, p1 was strongly associated with the compounds on the right side of the figure, indicating that higher concentrations in the samples have positive scores (the part with lower alcohol content). On the other hand, the compounds on the left side of the figure showed a negative correlation with p1, while the samples with lower alcohol content and negative scores for p1 corresponded with high concentrations of these compounds.

The PCA analysis showed that the changes in the aroma compounds in the same distillation system had a similar effect on the distillation time scale. This conclusion contributed to the establishment of limits for baijiu distillation cut points in the same distillation system. The common rules for aroma compounds are listed in Table S1. Many similar aroma compounds that changed belonged to a similar character due to their similar chemical properties. However, there were significant differences between the changes in the aroma compounds (Table S1). Too many changes occurred among the aroma compounds and the effects of these conflicts must be considered. This phenomenon made it difficult to select cut-off values for the recovery rate of the aroma compounds. In order to select the main representative aroma compounds, it was important to analyze the different character categories of the aroma compounds that changed during solid-state distillation.

**Figure 3:**
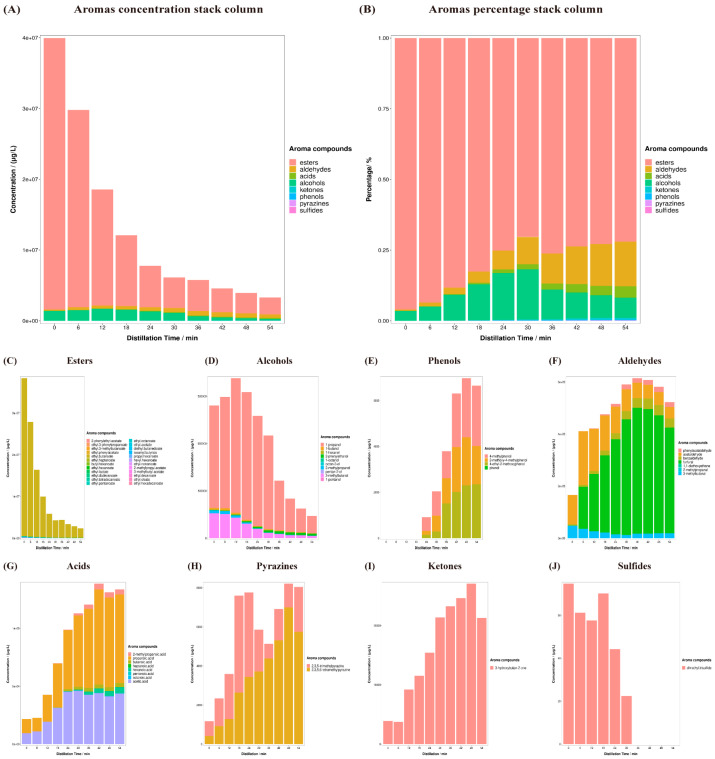
(**A**) Aroma compound concentration stack column of different categories’ proportions. (**B**) Aroma percentage stack column of different categories’ proportions. (**C**–**J**) Different aroma compound category (esters, alcohols, phenols, aldehydes, acids, pyrazines, ketones and sulfides) concentration stack columns.

**Categories of aroma compounds that varied and conflicted during distillation.** In order to evaluate the changes in the characteristics of the aroma compounds, these compounds were analyzed according to their different compound categories. Table S1 lists 57 aroma compounds and ethanol, for which the values are the average of the six distillations (three different fermentation pit layers in duplicate). The compounds could be divided into a total of eight categories: alcohols, aldehydes, esters, sulfides, acids, ketones, phenols and pyrazines. The Table S1 data were used to draw Figure 3. Figure 3A shows the total amount of these aroma compounds in the eight categories and the change in content during distillation is shown according to column stacking. Based on the total amount of aroma compounds, more aroma compounds were present in the early and final distillation stages. In the early distillation phase (0–18 min), esters and alcohols were the most important aroma compounds in the distillates. With an increasing distillation time, the content of aldehydes, acids and phenols gradually increased, and these became the most important aroma compounds. The different proportions of the individual categories are shown in Figure 3B. The proportion of esters and alcohols was >90% at the beginning of distillation, but toward the end of distillation, their proportion was reduced by that of other aroma compounds. Therefore, the distillates in the different distillation stages showed considerable differences in aroma character.

To understand the proportion of the aroma compounds in each group, the results in each category were presented in column stacks. As shown in Figure 3C, the abundance of esters gradually decreased, e.g., the ethyl acetate concentration decreased from 257,500 μg/L to 15,240 μg/L. However, not all esters showed similar change trends; for example, the concentration of ethyl lactate increased to 56,480 μg/L within 18 min and then decreased. The alcohols behaved similarly and the concentration of 1-pentanol differed from the others, as shown in Figure 3D. In contrast, the amount of phenols, aldehydes, acids, pyrazines and ketones gradually increased with the distillation time (Figure 3E–I). For example, the concentration of butanoic acid was below the detection limit at 0–12 min and gradually increased from 441 μg/L to 3683.3 μg/L by over 18–54 min. The concentration of 1,1-diethoxyethane decreased from 557.92 μg/L to 103.36 μg/L. The changes in the properties of the aroma compounds thus contradict each other in different categories. Even within the same category, there were conflicts with other substances for several aroma compounds due to their specific functional groups or functional structures. The changes in the aroma compounds showed similar trends between the compounds with similar chemical properties, such as acids or esters. Since compounds with the same chemical properties usually have a similar flavor character, the cut points should be set based on their chemical properties [33].

By analyzing the changes in concentration of the JXX baijiu aroma compounds, it was found that the difference in aroma compounds is more influenced by the characteristics of the aroma compounds than by the differences in the fermented grains. Different fermented JXX baijiu grains had similar physical structures although they contained different concentrations of aroma compounds [34]. Therefore, the thermodynamic conditions of the distillation system were similar for different fermented JXX baijiu grains. The mass transfer process of aroma compounds in the same thermodynamic environment was affected by their own thermodynamic properties. The thermodynamic properties were related to their chemical groups. Therefore, analyzing the change in the aroma compounds’ properties could help confirm the objectives by not only considering different categories of compounds but also selecting several compounds conflicting with others in the same categories.

### 3.2. Aroma Compound Kinetic Character in JXX Baijiu Solid-State Distillation

**Correlation analysis of the aroma compound changes.** The changes in the different categories of aroma compounds were analyzed. The results showed that the changes in many of the same categories of aroma compounds exhibited a similar trend. Therefore, a typical selection of aroma compounds could help to describe the changes in character and simplify the objectives of selecting cut-off values. Therefore, an appropriate selection criterion had to be developed. During the baijiu distillation process, the selection of cut-off points is usually based on the alcohol content and aroma compounds, and the character of the aroma is predicted by judging the alcohol content. Therefore, the relationship between the aroma compounds and ethanol concentration was important for the cut point selection for JXX baijiu distillation, as this relationship could serve as a criterion.

**Figure 4:**
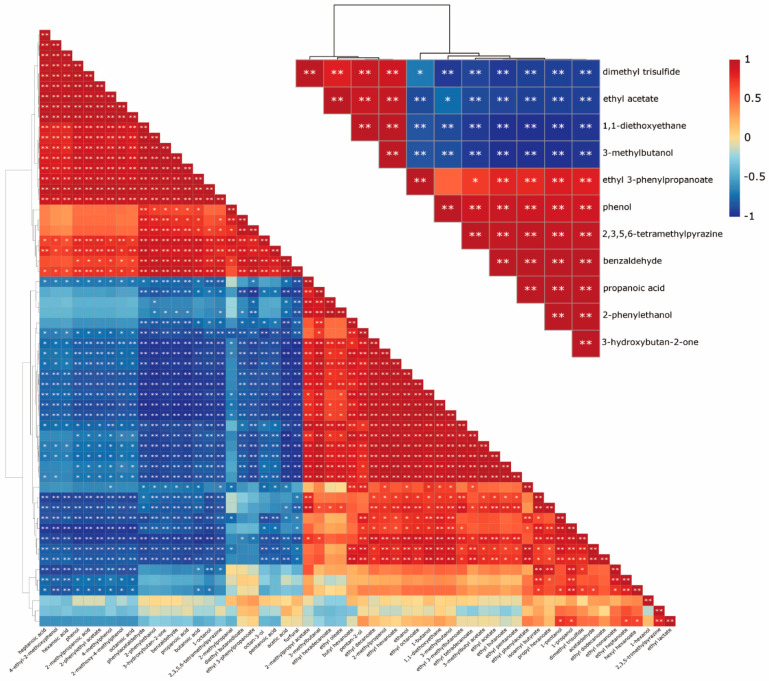
Heatmap of the Pearson correlation analysis. Values are Pearson’s correlation coefficients. * and ** denote correlation coefficients that are significant at the *p* < 0.05 and 0.01 levels, respectively.

As shown in Figure 4, the changes in the ethanol and 57 aroma compounds were analyzed using Pearson correlation analysis. Based on the correlation results, the aroma compounds could be divided into two groups, which is similar to the results of HCA analysis in Figure 2A. Several aroma compounds such as 3-methylbutanol, 1,1-diethoxyethane, ethyl acetate and dimethyl trisulfide showed high positive correlations with ethanol. Other aroma compounds such as propanoic acid, 2-phenylethanol, benzaldehyde, ethyl 3-phenylpropanoate, 3-hydroxybutan-2-one, phenol and 2,3,5,6-tetramethylpyrazine showed high negative correlations with ethanol. The high correlation of the aroma compounds could indicate a class change. Therefore, the representative aroma compounds could be selected based on their high positive and negative correlation values and the different chemical categories.

According to the correlation of the aroma compounds with ethanol, 11 aroma compounds were selected as representative targets. The correlation values of these aroma compounds are listed in Table 1. Among them, there were conflicts in the alcohols, aldehydes and esters for 3-methylbutanol and 2-phenylethanol; 1,1-diethoxyethane and benzaldehyde and ethyl acetate and ethyl 3-phenylpropanoate, respectively. These selected compounds could represent most of the changes in the aroma compounds.

Based on our previous studies, the ethanol solvent effect would cause different aroma compounds, such as ethyl acetate and propionic acid, to exhibit completely opposite changes [18]. These results showed that the changes in aroma compounds during distillation lead to conflicts. This phenomenon complicated the selection of cut-off values, as the improvement of a certain aroma compound led to the deterioration of other aroma compounds. Therefore, the description of the conflicting relationships between the aroma compounds was an important prerequisite for the regulation of the aroma compounds during distillation. These conflicting objectives had to be considered simultaneously when selecting the limit values of the cut points.

**Aroma compound kinetic character analysis.** When selecting the baijiu distillation cut points, it was difficult to directly regulate the changes in aroma compounds. Since all the aroma compounds were distilled at the same time, the selection of cut point times was determined with relatively high concentrations. Control over the baijiu aroma was achieved by selecting the target aroma compounds that had a relatively higher distillation efficiency than the other compounds. Thus, the efficiency of aroma compounds was an important indicator reflecting the character of changes and conflicts.

Based on the changes in the aroma compounds, a representative pseudo-first-order kinetic model could be created for the aroma compounds. The changes in the characters of the aroma compounds during distillation could be described using this model. As shown in Table 1, 11 selected aroma compounds that change their character could be compared based on the kinetic parameter K. 3-methylbutanol, 1,1-diethoxyethane, ethyl acetate and dimethyl trisulfide kinetic showed positive values for K, which means that their change character was a gradual decrease in concentration changes. In contrast, propanoic acid, 2-phenylethanol, benzaldehyde, ethyl 3-phenylpropanoate, 3-hydroxybutan-2-one, phenol and 2,3,5,6-tetramethylpyrazine showed negative values for K, which means that their change character constitutes a gradual increase in concentration changes. In addition, the K values differed; for instance, 2,3,5,6-tetramethylpyrazine and phenol were −0.03668 and −0.0627, respectively. Therefore, there were conflicts in different categories of aroma compounds.

**Figure 5:**
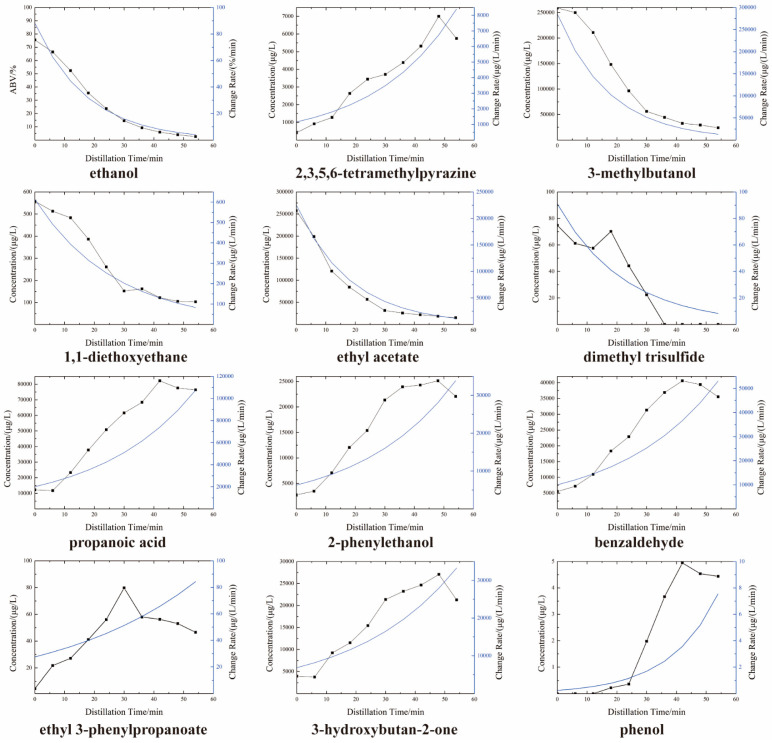
Aroma compound concentration change with distillation time (black lines); aroma compound concentration change rates with distillation time (blue lines).

To describe this conflict directly, the amount of aroma compounds that have accumulated at a given time can be calculated using Equation (2). As shown in Figure 5, the change in the distillation rate of the aroma compounds could be compared with the distillation time (see blue lines). The distillation rate of the JXX baijiu characteristic aroma compound 2,3,5,6-tetramethylpyrazine gradually increased from 1160 μg/(L/min) to 8438.6 μg/(L/min). The distillation rates of 3-methylbutanol, 1,1-diethoxyethane, ethyl acetate and dimethyl trisulfide gradually decreased as the effect of ethanol as a solvent diminished. The different chemical properties of these compounds also affected their distillation rates. If 2,3,5,6-tetramethylpyrazine was to be enhanced as the distinctive character aroma compound of sauce aroma baijiu, the distillation rates of 1,1-diethoxyethane, ethyl acetate and dimethyl trisulfide were in contradiction. At the same time, propanoic acid, 2-phenylethanol, benzaldehyde, ethyl 3-phenylpropanoate, 3-hydroxybutan-2-one and phenol showed different rates of change than 2,3,5,6-tetramethylpyrazine, although the trends were consistent. In the solid-state distillation of JXX baijiu, the mass transfer of aroma compounds was affected by heat transfer and the solvent ethanol [18]. Ethanol as a solvent improved the screening and distillation efficiency of the aroma compounds. Therefore, different categories of aroma compounds had significantly different characters and rates from each other.

The changes in the character of the compounds during distillation could be described using the kinetic parameter K [35]. By describing the changes, the efficiency of the distillation of the aroma compounds can be analyzed. When selecting the limits for JXX baijiu distillation, an improvement in the recovery of certain aroma compounds leads to the deterioration of other aroma compounds where the alteration characteristics are in conflict. Therefore, the aroma compound change characters quantified supported basic cut point selection optimization. In addition, the conflicts between them could be found according to their different distillation rates with the distillation time. In order to regulate the distinctive aroma compound 2,3,5,6-tetramethylpyrazine of JXX baijiu and improve the JXX aroma character, the conflicts with other aroma compounds had to be resolved.

### 3.3. JXX Baijiu Distillation Time Cut Point Selection

**Selecting JXX baijiu distillation time cut points based on recovery rate.** The concentration balance between the aroma compounds is important, as excessive enhancement of one or more flavorings can unbalance the baijiu aroma. Therefore, the thresholds for enhancing the distinctive aroma character within an appropriate range are important to improve the identification of baijiu. Cut points usually consider the targets for the different aroma compounds and multi-objective optimization (MOO) is suitable to solve this problem. Solving a MOO problem involves a set of optimal points known as a non-dominated solution or Pareto front (PF), and its optimal decision variables are known as a Pareto set [36]. The distillation of JXX baijiu involves many objectives with aroma characters that need to be optimized simultaneously and these objectives may conflict with each other, i.e., the improvement of one objective leads to the deterioration of another objective. This means that the selection of baijiu distillation cut points for different aroma characters is better carried out within an MOO approach [37]. The cut point design of the distillation limits for a brandy Charentais alembic has been shown to be suitable for the selection of distillation aromas [7]. This is particularly evident when multiple compounds need to be considered when setting limits and intuitive changes are not helpful.

For the selection of baijiu distillation cut points that enhance the distinctive character of the aroma compounds, the MOO model was used to determine the optimal cut time under different boundary conditions. Since all aroma compounds were distilled simultaneously, the change in aroma compounds could not be used to develop the model. Therefore, a kinetic model of the aroma compounds was used. The recovery rates were calculated for the selection of the optimal distillation time cut points.

**Figure 6:**
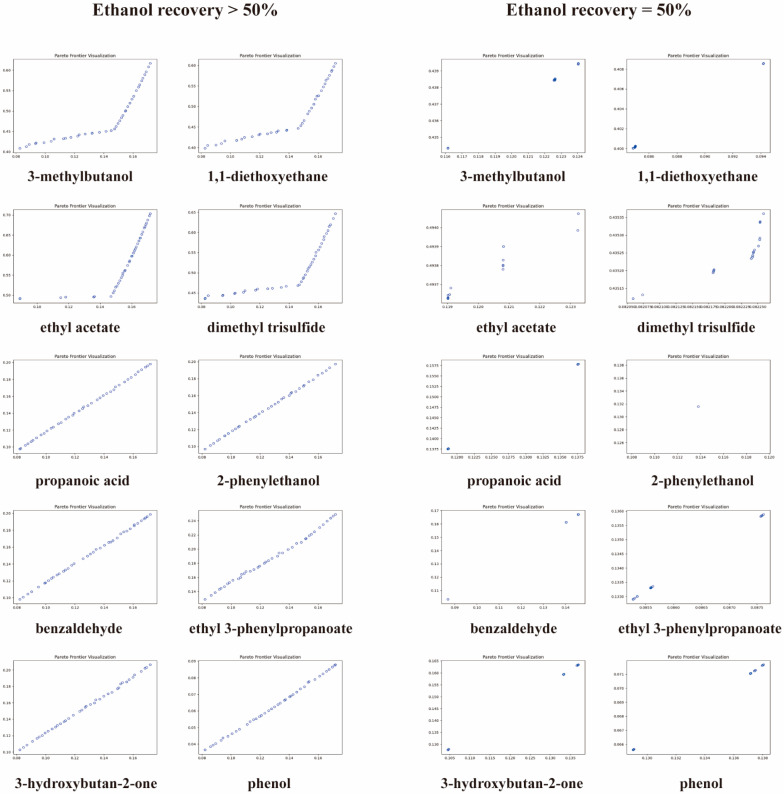
The Pareto front of 2,3,5,6-tetramethylpyrazine and other aroma compounds (3-methylbutanol, 1,1-diethoxyethane, ethyl acetate, dimethyl trisulfide, propanoic acid, 2-phenylethanol, benzaldehyde, ethyl 3-phenylpropanoate, 3-hydroxybutan-2-one and phenol) in ≥50% ethanol recovery rate constraints.

In the MOO model, 2,3,5,6-tetramethylpyrazine was optimized to achieve a maximum recovery rate, while at the same time, 1 out of 10 typical aroma compounds should achieve the minimum recovery rate. Two different boundary conditions were defined, which were ≥50% of the recovery rate of ethanol. The results are shown in Figure 6. With an inequality condition of ≥50% of the ethanol recovery rate, there were a number of non-dominating solutions or PFs for each intersection of 2,3,5,6-tetramethylpyrazine and the typical aroma compounds. The PF meant that each range of the ethanol recovery rate represented an optimal selection of cut points. As the recovery rate of ethanol increased, the recovery rate of 2,3,5,6-tetramethylpyrazine and another aroma compound also increased simultaneously. Therefore, similar limits were set for 3-methylbutanol, 1,1-diethoxyethane, ethyl acetate and dimethyl trisulfide due to their increasing tendencies. In terms of the recovery rate, 2,3,5,6-tetramethylpyrazine was found to have a higher rate of increase, at ~70% of the ethanol recovery rate, than the others. In addition, propanoic acid, 2-phenylethanol, benzaldehyde, ethyl 3-phenylpropanoate, 3-hydroxybutan-2-one and phenol did not differ significantly in their inequality constraints. Therefore, there was a non-dominated solution for the equality constraint of the specific ethanol recovery rate for each limit. At <70% of the ethanol recovery rate, it was more favorable to increase the 2,3,5,6-tetramethylpyrazine recovery rate.

**Figure 7:**
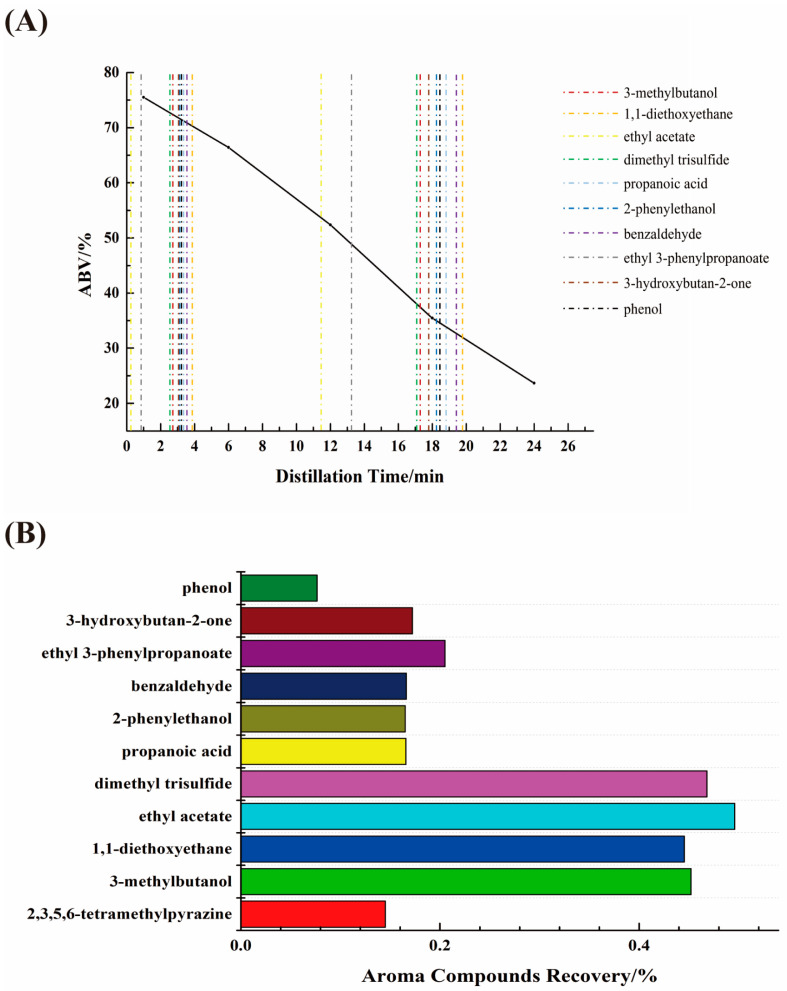
(**A**) Visualization results of JXX baijiu solid-state distillation time cut points of 2,3,5,6-tetramethylpyrazine with other aroma compounds. (**B**) Aroma compound recovery rate at optimum distillation time cut points.

The cut points at 50% of the ethanol recovery rate are plotted in Figure 6 to discuss the cut points in terms of their equality conditions. The results show that the cut point for each aroma compound was reduced to a small range. Therefore, the optimal solution can be considered the only one in the equality constraint. All the optimal cut points are listed in Table S2. All the first cut points were at 2.6–3.9 min and the second cut points were at 17.3–19.8 min, except for ethyl acetate and ethyl 3-phenylpropanoate. The cut points of 2,3,5,6-tetramethylpyrazine with ethyl acetate or ethyl 3-phenylpropanoate did not match the actual product situation. Therefore, the cut times for the optimal cut points were similar, and are shown in Figure 7A. The first cut point was aligned with a 72% ethanol volume concentration and the second cut point with a 35% ethanol volume concentration.

**Selection of the best cut points for recovery rates.** To maximize the improvement in the 2,3,5,6-tetramethylpyrazine recovery rate, the distillation cut points of 2,3,5,6-tetramethylpyrazine and 1,1-diethoxyethane were chosen so that the 2,3,5,6-tetramethylpyrazine recovery rate could reach a maximum of 14.53%. The recovery rates of the other aroma compounds are shown in Figure 7B. The recoveries of 3-methylbutanol, 1,1-diethoxyethane, ethyl acetate and dimethyl trisulfide were 45.19%, 44.54%, 49.58% and 46.80%, respectively. The recoveries of propanoic acid, 2-phenylethanol, benzaldehyde, ethyl 3-phenylpropanoate, 3-hydroxybutan-2-one and phenol were 16.58%, 16.51%, 16.63%, 20.51%, 17.21% and 7.65%, respectively. These limits were selected on the basis of a lower recovery rate.

By selecting the MOO method, suitable JXX baijiu distillation time cut points was proposed to enhance the distinctive aroma character within a reasonable recovery rate range. This method could also be applied to other forms of baijiu solid-state distillation aroma character regulation or other aroma character improvements. The selection of distillation timing was an important method for regulating the baijiu aroma compounds. But the accuracy and stability of regulation require further aroma compound mass transfer prediction and mechanism model development.

## 4. Conclusions 

The JXX baijiu aroma compound change characters during solid-state distillation were different and mainly had two contrary trends. The analysis results showed that the changes in the aroma compounds were more affected by the characteristics of the aroma compounds than by the differences in the fermented grains. Then, the kinetic model described two opposite changes based on positive or negative kinetic parameters. By analyzing the kinetic model and the MOO model, a method was used to regulate the distillation of the aroma compounds to increase the proportion of the 2,3,5,6-tetramethylpyrazine recovery rate to improve the distinctive character of the JXX baijiu. This study on the change in aroma compounds formed the basis for the investigation of the mass transfer mechanisms in the solid-state distillation of baijiu, as well as aroma compound regulation in distillation.

## Figures and Tables

**Table 1:** Aroma compound categories, revaluation with ethanol, kinetic parameters with distillation process of aroma compounds.

Aroma Compounds	Group	Revaluation with Ethanol	C_e_	K
2,3,5,6-tetramethylpyrazine	pyrazines	−0.945318672	−31,599.88	−0.03668
3-methylbutanol	alcohols	0.995366909	7,306,350.00	0.03898
1,1-diethoxyethane	aldehydes	0.985116613	16,578.67	0.037
ethyl acetate	esters	0.975771218	4,101,260.00	0.055
dimethyl trisulfide	sulfides	0.901823171	2054.20	0.04423
propanoic acid	acids	−0.981964632	−648,421.10	−0.03102
2-phenylethanol	alcohols	−0.982819036	−200,920.97	−0.03122
benzaldehyde	aldehydes	−0.973942394	−323,923.35	−0.03089
ethyl 3-phenylpropanoate	esters	−0.855488216	−1323.86	−0.02075
3-hydroxybutan-2-one	ketones	−0.969204139	−233,640.61	−0.02929
phenol	phenols	−0.847540024	−4.08	−0.0627

## Data Availability

Data is contained within the article or Supplementary Materials.

## Supplementary Materials

The following supporting information can be downloaded at: https://www.mdpi.com/article/10.3390/foods13020232/s1. Table S1. Aroma compounds concentration (μg/L) change in distillation. Table S2. Cut points in distillation recipes of 2,3,5,6-tetramethylpyrazine with other aroma compounds.
