# Supplementary material for: Aroma Compound Changes in the Jiangxiangxing Baijiu Solid-State Distillation Process: Description, Kinetic Characters and Cut Point Selection
Source: Foods. 2024 Jan 11;13(2):232. doi: 10.3390/foods13020232 (PMC10814311; doi:10.3390/foods13020232)
Supplement: Supplementary file 1 [file foods-13-00232-s001.zip › foods-2795814-supplementary.pdf]

Supplementary Information

AROMA COMPOUNDS CHANGES OF JIANGXIANGXING BAIJIU SOLID-STATE DISTILLATION PROCESS : DESCRIPTION, KINETIC CHARACTERISTICS AND CUT POINTS SELECTION

Yuchen Gao[a], Qiang Yang[b], Guangyuan Jin[a], Shengzhi Yang[b], Ruiyang Qin[a], Linjie Lyu[b], Xianze Yao[b], Rongzhen Zhang[a], Shuang Chen[a] and Yan Xu[a]*

[a] Lab of Brewing Microbiology and Applied Enzymology, Key Laboratory of Industrial Biotechnology of Ministry of Education, State Key Laboratory of Food Science & Technology & School of Biotechnology, Jiangnan University, Wuxi 214122, Jiangsu, China

[b] Jing Brand Co., Ltd, HuangShi, HuBei 435100, China

* corresponding author: yxu@jiangnan.edu.cn

Table S1. Aroma compounds concentration (μg/L) change in distillation

No.	Aroma Compounds	0min	6min	12min	18min	24min	30min	36min	42min	48min	54min
1	ethanol[a]	75.52	66.42	52.37	35.45	23.67	14.50	9.18	5.93	3.98	2.58
2	2-methylpropanoic acid[f]	nd	nd	nd	223.07	362.00	1974.18	3666.17	4945.75	4536.95	4437.21
3	propanoic acid[f]	12187.28	11741.00	23282.90	37787.14	50815.98	61545.99	68393.18	82169.89	77534.73	76354.57
4	butanoic acid[f]	nd	nd	nd	441.00	1954.68	2463.73	3213.81	3457.41	3377.71	3683.30
5	heptanoic acid[s]	nd	nd	nd	nd	nd	9.07	11.04	20.84	24.94	26.46
6	hexanoic acid[f]	nd	nd	nd	nd	298.44	663.90	2562.87	3761.96	4204.12	5239.99
7	pentanoic acid[s]	16.35	1.98	3.30	19.63	78.98	219.33	130.41	151.97	142.11	177.43
8	octanoic acid[f]	nd	nd	nd	nd	0.83	4.74	6.02	8.83	11.65	12.07
9	acetic acid[f]	9486.95	10959.30	19409.54	31446.05	45295.86	46132.44	42628.29	44125.56	41401.52	43728.14
10	1-propanol[f]	1081113.65	1175681.55	1421463.11	1348806.64	1163167.78	990802.95	526167.84	349863.54	250587.37	183747.65
11	1-butanol[f]	19042.84	20751.83	17229.24	10720.45	5708.06	4970.84	3654.77	2445.39	2181.49	1081.70
12	1-hexanol[f]	3708.69	6572.66	5628.25	4854.21	4271.48	7259.51	6496.18	6355.74	3667.78	2631.64
13	2-phenylethyl acetate[f]	nd	nd	nd	223.07	362.00	1974.18	3666.17	4945.75	4536.95	4437.21
14	ethyl 3-phenylpropanoate[s]	4.72	21.76	27.17	41.19	56.12	79.79	58.03	56.29	53.16	46.59
15	phenylacetaldehyde[s]	nd	319.92	1339.88	4496.84	11297.72	18662.16	17469.86	18211.97	20110.76	18554.07
16	acetaldehyde[f]	112918.17	206289.69	162624.65	134327.14	101582.54	84257.37	59285.76	52699.81	50014.81	43952.36
17	ethyl 3-methylbutanoate[f]	10049.51	7201.11	5391.24	3794.34	3616.68	3447.17	3032.58	2099.64	1639.59	1207.14
18	benzaldehyde[f]	5521.49	7178.54	10927.56	18331.11	22884.20	31317.67	36877.83	40591.29	39409.78	35522.71
19	2-phenylethanol[f]	2753.52	3473.51	7080.91	12048.58	15387.29	21346.33	23943.27	24289.26	25129.14	22079.49
20	ethyl phenylacetate[f]	211.84	9363.00	6599.84	3663.65	894.08	597.77	541.39	nd	nd	nd
21	ethyl butanoate[f]	37867951.60	27505790.74	16094245.43	9765340.98	5703925.20	4212858.30	4313695.47	3301365.26	2799594.36	2325769.98
22	ethyl heptanoate[s]	2.58	4.75	3.23	4.77	5.69	4.28	2.81	2.13	2.01	1.30
23	butyl hexanoate[f]	307.09	583.08	272.60	nd	nd	nd	nd	nd	nd	nd
24	ethyl hexanoate[f]	51492.06	43667.26	30842.41	21670.07	14413.02	7156.35	8272.89	6316.03	6070.21	5330.91
25	furfural[f]	0.00	161564.88	220332.99	296495.40	365511.46	444466.91	484220.83	478324.81	453575.01	405808.85
26	ethyl lactate[f]	14847.04	27942.63	48179.02	56476.27	43471.19	30683.96	25562.49	21165.05	20708.62	19342.10
27	ethyl dodecanoate[s]	29.17	60.86	44.27	21.80	22.12	16.32	11.56	3.11	3.01	1.87
28	ethyl tetradecanoate[f]	1269.39	1242.93	867.29	225.70	nd	nd	nd	nd	nd	nd

29	ethyl pentanoate[f]	4179.12	3099.68	1859.50	1520.03	759.74	491.27	514.87	466.02	451.23	408.93
30	ethyl octanoate[f]	38346.86	28473.23	27676.93	20288.41	11925.52	8735.33	5765.48	4318.08	3261.98	3140.49
31	ethyl acetate[f]	257495.05	198847.89	120702.96	84447.80	56659.09	31607.87	25799.83	21889.92	18698.48	15236.24
32	1,1-diethoxyethane[f]	557.92	512.70	483.42	386.68	261.16	152.45	161.93	121.79	105.54	103.36
33	1-octanol[s]	0.45	19.22	27.73	41.03	63.91	92.06	127.34	153.01	161.13	164.39
34	diethyl butanedioate[s]	9.66	139.47	242.46	583.21	1160.04	1831.78	1087.75	967.98	978.23	850.34
35	isoamyl butyrate[s]	14.38	13.71	9.19	10.39	14.68	11.16	5.83	4.35	2.53	2.59
36	propyl hexanoate[s]	23.88	18.37	10.72	13.35	17.46	14.23	7.29	4.08	3.87	2.97
37	hexyl hexanoate[s]	0.27	0.50	0.37	0.63	0.36	0.60	0.13	0.07	0.13	0.03
38	ethyl nonanoate[s]	87.18	71.63	70.90	84.45	115.72	90.78	52.36	28.78	24.32	17.46
39	2-methylpropyl acetate[s]	363.53	167.55	101.59	86.67	125.44	112.21	65.03	35.64	14.36	11.10
40	3-methylbutyl acetate[f]	28455.76	21709.40	14708.74	11202.41	4666.13	3310.23	3252.19	2672.77	2664.46	2427.40
41	ethyl decanoate[f]	34858.36	22940.62	23879.63	17142.33	8519.85	3698.75	2915.80	2422.99	2799.33	2773.72
42	2-methylpropanal[s]	184.70	105.37	69.14	90.18	267.49	359.23	248.39	240.08	249.09	194.09
43	octan-3-ol[s]	86.75	75.55	62.76	82.26	156.87	168.77	176.39	181.68	177.60	135.35
44	2-methylpropanol[f]	32130.46	30373.49	21904.89	13607.54	5805.51	3791.65	3008.82	2407.41	1951.31	1493.51
45	pentan-2-ol[f]	2608.17	3196.54	4399.25	1847.02	nd	nd	nd	nd	nd	nd
46	3-methylbutanol[f]	259081.65	249884.62	210817.38	148260.51	96442.24	56199.35	44510.10	32593.10	29450.52	24119.73
47	3-methylbutanal[f]	47167.42	35538.10	26043.79	21427.45	14254.87	11572.97	16711.39	17240.53	18423.33	18422.27
48	1-pentanol[f]	1562.24	2660.78	3302.13	3296.45	1658.08	682.12	177.03	nd	nd	nd
49	ethyl oleate[f]	17246.43	2804.83	2985.25	750.77	109.77	nd	nd	nd	nd	nd
50	ethyl hexadecanoate[f]	43893.85	8551.01	9961.14	2943.05	1599.92	3224.96	3851.37	1800.61	1357.59	4224.10
51	3-hydroxybutan-2-one[f]	3912.64	3747.08	9229.27	11522.84	15435.26	21372.54	23231.50	24640.94	27065.67	21280.53
52	2,3,5-trimethylpyrazine[f]	765.60	1422.62	2310.32	4960.84	4321.46	2150.09	752.17	1599.89	1211.12	2296.18
53	2,3,5,6-tetramethylpyrazine[f]	410.38	918.11	1279.15	2639.19	3443.14	3713.25	4384.03	5314.25	7000.22	5747.23
54	4-methylphenol[s]	nd	nd	nd	nd	596.91	1062.29	1177.04	2337.21	2580.40	2631.95
55	2-methoxy-4-methylphenol[s]	nd	nd	nd	nd	172.34	691.43	1103.27	1957.20	2094.64	1666.69
56	4-ethyl-2-methoxyphenol[s]	nd	nd	nd	nd	147.99	284.99	1506.63	2009.41	2299.79	2346.09
57	phenol[s]	nd	nd	nd	0.22	0.36	1.97	3.67	4.95	4.54	4.44
58	dimethyl trisulfide[s]	74.77	61.21	57.61	70.21	44.27	22.42	nd	nd	nd	nd

[a]ethanol means alcohol content (ABV, %). [f]Quantified by GC-FID. [s]Quantified by HS-SPME-GC-MS. nd: no detected.

Table S2. Cut points in distillation recipes of 2,3,5,6-tetramethylpyrazine with other aroma compounds

Optimization objects	First cut/min	Second cut/min	2,3,5,6-tetramethylpyrazine recovery/%	Other aroma compounds recovery/%
3-methylbutanol	2.8	17.3	12.29	14.24
1,1-diethoxyethane	3.9	19.8	14.53	44.56
ethyl acetate	0.2	11.4	8.24	49.02
dimethyl trisulfide	2.6	17.1	12.08	45.73
propanoic acid	3.4	18.8	13.64	15.66
2-phenylethanol	3.2	18.2	13.15	15.06
benzaldehyde	3.6	19.4	14.15	16.25
ethyl 3-phenylpropanoate	0.9	13.2	9.33	14.31
3-hydroxybutan-2-one	3.1	17.8	12.69	15.23
phenol	3.3	18.5	13.39	6.88
